# Incidence, Risk Factors and Outcomes of Kidney and Liver Cyst Infection in Kidney Transplant Recipient With ADPKD

**DOI:** 10.1016/j.ekir.2022.01.1062

**Published:** 2022-02-03

**Authors:** Charles Ronsin, Anis Chaba, Ondrej Suchanek, Jean-Philippe Coindre, Clarisse Kerleau, Claire Garandeau, Aurélie Houzet, Diego Cantarovich, Jacques Dantal, Gilles Blancho, Magali Giral, Grégoire Couvrat-Desvergnes, Simon Ville

**Affiliations:** 1 Department of Nephrology and Immunology, Center Hospitalier Universitaire de Nantes, Nantes, France; 2 Paris University, Paris, France; 3 Cambridge University Hospitals NHS, Foundation Trust, NIHR Cambridge Biomedical Research Centre, Cambridge, UK; 4 Department of Nephrology, Le Mans Hospital, Le Mans, France; 5 Centre de Recherche en Transplantation et Immunologie UMR 1064, Institut National de la Santé et de la Recherche Médicale (INSERM), Université de Nantes, Nantes, France; 6 Department of Nephrology, Dialysis and Transplantation, Departmental Hospital of Vendée, La Roche-sur-Yon, France

**Keywords:** autosomal dominant polycystic kidney disease, cyst infection, incidence, kidney transplantation, risk factors

## Abstract

**Introduction:**

Cyst infection is a known complication of autosomal dominant polycystic kidney disease (ADPKD). Here, we describe incidence, risk factors, clinical presentation, and outcomes of cyst infection in kidney transplant recipient.

**Methods:**

We conducted a single-center retrospective cohort study of patients with ADPKD with renal allografts between January 1, 2009, and October 31, 2020. Cyst infection diagnosis was based on previously described clinical and radiological criteria, using positron emission tomography when available.

**Results:**

A total of 296 patients with ADPKD with renal allografts were included, and 21 patients experienced 22 episodes of cyst infection over a median follow-up of 4 (2–7) years. The cumulative incidence rate was 3% at 1 year, 6 % at 5 years, and 12% at 10 years after transplantation. In multivariate analysis, history of cyst infection before transplantation was the only significant risk factor identified to predict the occurrence of cyst infection after kidney transplantation (hazard ratio [HR] 3.47, 95% CI 1.29–9.31). The clinical presentation at diagnosis of cyst infection included isolated fever in 5 (23%) episodes, acute kidney injury in 12 (55%), and severe sepsis/septic shock in 3 (14%) episodes. Among the 16 (73%) episodes with culture positivity, *Escherichia coli* was the most common pathogen. There was no difference between early (≤1 year after transplantation) and late (>1 year) cyst infection episodes in terms of clinical presentation and outcomes. Cyst infection was significantly associated with graft loss (HR 3.93, 95% CI 1.21–12.80), but no causal relationship could be established.

**Conclusion:**

Incidence of cyst infection in ADPKD after kidney transplantation is low, history of cyst infection representing the main risk factor.

## Graphical abstract

**Figure:**
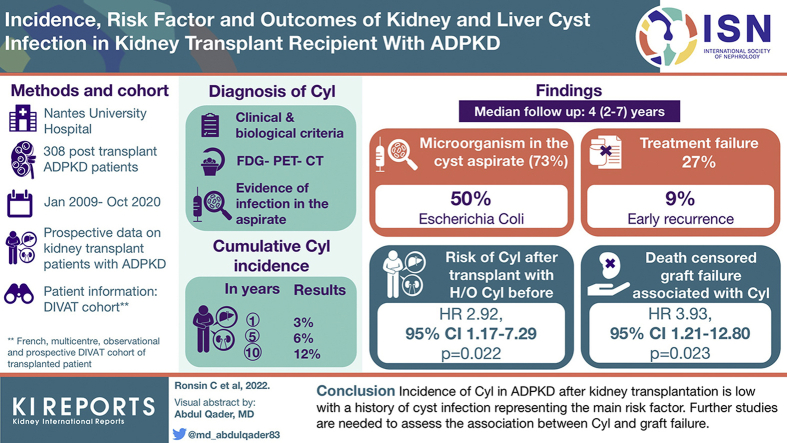

ADPKD is the most prevalent hereditary kidney disease accounting for approximately 5% to 8%^1^, ^2^, ^3^, ^4^ of patients with end-stage kidney failure in Western countries. Despite the emergence of new treatments slowing down the progression of ADPKD,^5^, ^6^, ^7^ half of the patients aged >60 years develop kidney failure.^4^^,^^8^ For these patients, kidney transplantation is the most frequently used renal replacement therapy^9^ in Europe.^10^

Apart from the development of kidney failure, one of the well-known complications of ADPKD is a kidney or liver cyst infection, the incidence of which, in patients with or without renal replacement therapy is estimated around 1 per 100 person-years.^11^ History of cyst infection has been reported as risk factor for cyst infection in chronic dialysis patients,^12^ while others such as female sex, age, and recent instrumentation in the urinary tract have been suspected.^13^ The diagnosis of cyst infection has been recently improved with the wide use of ^18^F-fluorodeoxyglucose positron emission tomography/computed tomography (FDG-PET/CT).^14^, ^15^, ^16^ However, the antibiotic treatment failure and/or infection relapse is not uncommon and, in some circumstance, requires a percutaneous or surgical drainage.

Kidney transplantation together with its associated immunosuppression could represent an important risk factor for cyst infection in ADPKD. To date, no studies have investigated the incidence, risk factors, clinical presentation, and outcomes of cyst infection in ADPKD specifically after kidney transplantation. We addressed this gap of evidence by retrospectively studying a well-characterized large cohort of kidney transplant recipients with ADPKD.

## Methods

### Study Population and Available Data

We conducted a single-center study on all consecutive adult patients transplanted or retransplanted at Nantes University Hospital between January 1, 2009, and October 31, 2020, for whom the primary renal disease was ADPKD. Follow-up was finished on March 31, 2021.

Patients with primary nonfunctioning allograft were excluded from the study. All data were extracted from the French multicenter, observational, and prospective DIVAT cohort of transplanted patients (www.divat.fr). The reported clinical and research data are in line with the Principles of the Declaration of Istanbul on Organ Trafficking and Transplant Tourism. The DIVAT cohort received CNIL (Commission Nationale de l’Informatique et des Libertés) (No914184) and the CCTIRS (Advisory Committee on Information Processing in Material Research in the Field of Health) approval.

Regarding cyst infection episodes, although the infectious episodes were recorded prospectively, each medical records of the patients included in the study were retrospectively reviewed to identify patient with history of cyst infection before transplantation and during the post-transplantation period. Furthermore, clinical, biological, imaging, and cyst infection parameters and cyst infection outcomes were collected. Corticosteroid exposure was calculated using prescription data collected in parallel in our electronic system. Each prescription was reviewed in terms of start (excluding intravenous steroids used concomitantly with thymoglobulin antibodies) and stop (censored to death, graft failure, last follow-up, or cyst infection).

### Definition of Cyst Infection

Diagnostic criteria for liver or native kidney cyst infection were based on Sallée et al.^11^ with the implementation of FDG-PET-CT.^11^^,^^13^, ^14^, ^15^ Cyst infection was labeled as definite when confirmed by cyst aspiration showing evidence of infection (neutrophils debris and/or microorganism); cyst infection was probable if either (i) all the following features were present: fever, abdominal or flank pain, C-reactive protein >50 mg/l, and the absence of any significant recent intracystic bleed (based on abdominal computed tomography) or other causes of fever or (ii) FDG-PET-CT supported the diagnosis of cyst infection (increased cyst wall metabolism or intracystic 18-FDG uptake) and C-reactive protein >50 mg/l without an alternative cause for an inflammatory response.

Clinical and biological terms used in this study were defined as follows: typical cyst infection presentation was defined as the presence of abdominal/flank or lumbar pain and fever >38.5 °C; atypical presentation was defined by the lack of fever and/or isolated fever without abdominal/back or flank pain, and thus, probable cyst infection in patients with atypical presentation was diagnosed on the basis of PET-CT as outlined earlier. Acute kidney injury was defined using Acute Kidney Injury Network criteria.^17^ Severe sepsis and septic shock definitions were based on the third international consensus definition for sepsis.^18^ Radiologic criteria for cyst infection were met if either (i) kidney and liver ultrasound demonstrated at least 1 cyst with debris and thickened wall and/or distal acoustic enhancement or/and (ii) CT scan showed enhanced wall thickness and/or pericystic fat infiltration in at least 1 cyst and PET-CT showed higher 18F-FDG uptake in cyst walls or within the cyst compared with surrounding parenchyma. Treatment failure was defined as persistence or increase of C-reactive protein or fever and the need for modification of antibiotic therapy (i.e., increasing doses or switching to broader spectrum antibiotic) or cyst drainage. Early recurrence was defined as cyst infection recurrence within 3 months after the discontinuation of antibiotic therapy.

### Statistical Analysis

We considered each transplantation as a statistical unit. Continuous variables were summarized as means with SD, and categorical and ordinal variables were summarized as frequencies and percentages. Missing data were removed from percentages calculation.

The cumulative incidence curve of cyst infection was obtained using the reverse Kaplan–Meier estimator.

Univariate analysis (logrank test) was used to identify possible risk factors. A multivariable backward selection procedure was implemented, with a univariate threshold *P* < 0.20 for inclusion, and a *P* < 0.05 was considered statistically significant in the final model. Although not significant in univariate analysis, corticosteroids exposure and age were forced into the final multivariable model because of their known impact on infection incidence. Given the paucity of event (cyst infection) in our cohort, few clinical covariables were analyzed in a multivariable approach. Baseline covariables in the model included age, sex, history of diabetes, history of cardiovascular event, history of nephrectomy, body mass index, cold ischemia, delayed graft function, type of donor (living, standard criteria, or extended criteria), and lymphocyte depleting or nondepleting treatment in the induction regimen. Corticosteroid exposure ≥ 1 year before cyst infection and the absence or presence of corticosteroids treatment during the follow-up were also included in the analysis.

For death-censored graft survival analysis, de novo donor specific antibodies and rejection were included in the follow-up data only if they appeared after the first cyst infection episode.

*P* < 0.05 was considered as statistically significant. All analyses were performed using the (1.4 1717) version of the R software.

## Results

### Study Population Characteristics

In total, 308 kidney transplants were performed between January 2009 and October 2020 in patients with ADPKD. After the exclusion of patients with primary allograft nonfunction (*n* = 12), 296 patients were recruited in the study. Eight patients had 2 transplants during the study period (first and second [*n* = 7], second followed by a third [*n* = 1]).

**Figure 1:**
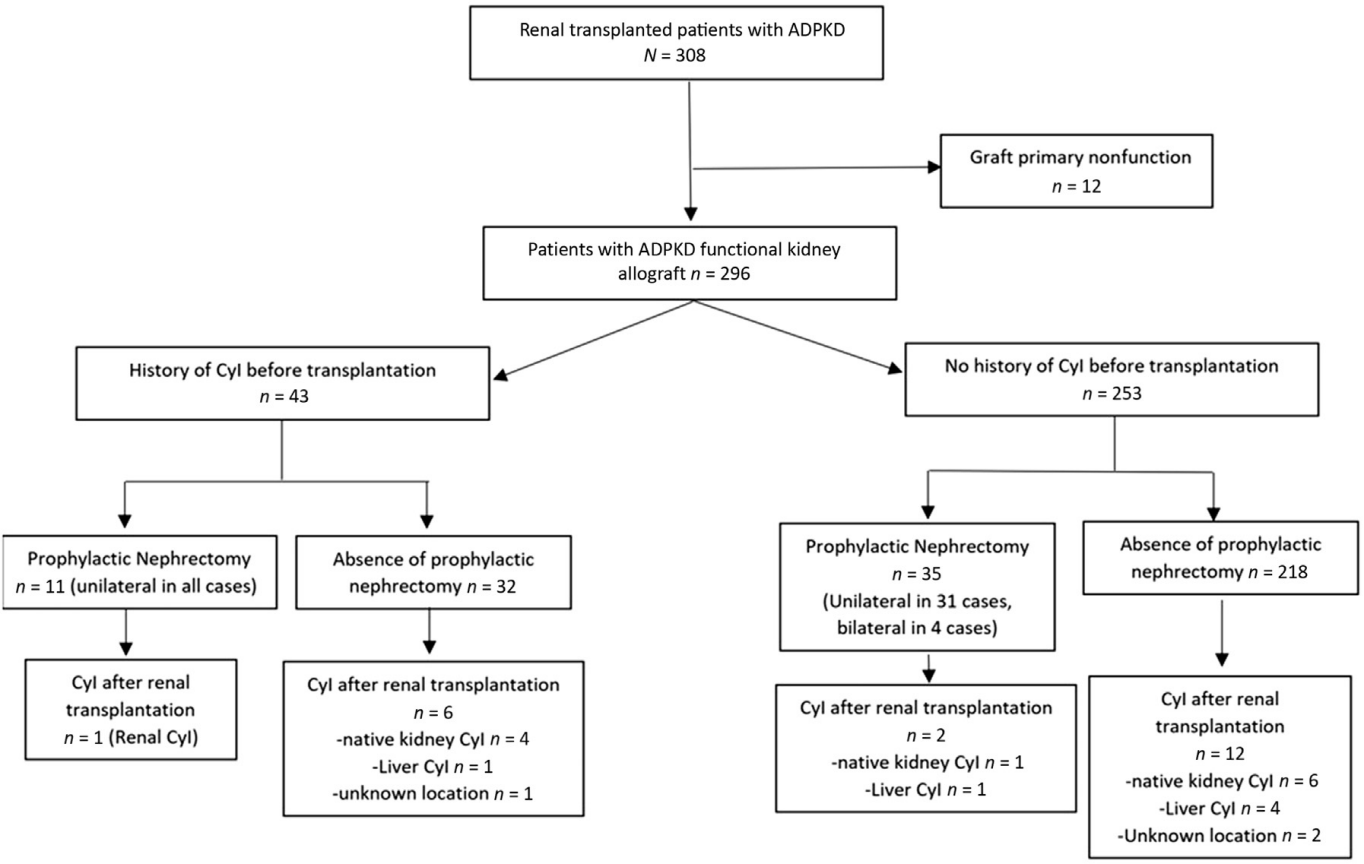
Flow chart of patients with ADPKD with kidney allograft according to the presence of cyst infection before the transplantation. ADPKD, autosomal dominant polycystic kidney disease; Cyl, cyst.

A total of 61 patients (21%) received an organ from a living donor and 235 (79%) from a deceased donor (Table 1). Mean recipient age was 60 ± 8 years, 53% were male, and mean recipient body mass index was 26 ± 4 kg/m^2^. Before transplantation, 182 recipients (61%) were on chronic dialysis, 27 (9%) had diabetes, 43 (15%) experienced cyst infection, 42 (14%) had unilateral nephrectomy, and 4 (1%) had bilateral nephrectomy (all of them had liver cyst). Indications for native nephrectomy included insufficient room for renal allograft (*n* = 25, 54%), recurrent cyst infection (*n* = 6, 13%), recurrent bleeding (*n* = 6, 13%), severe pain (*n* = 4, 9%), suspected malignancy (*n* = 2, 4%), and unknown (*n* = 3, 7%). By the time of transplant surgery, 11 patients (26%) with a history of cyst infection had unilateral nephrectomy (for recurrent cyst infection in *n* = 6 and insufficient room for renal allograft in *n* = 5) (Figure 1).

**Table 1:** Characteristics at the time of renal transplantation of 296 patients with ADPKD and functional renal allografts according to the presence of native kidney or liver cyst infection during follow-up

Characteristics	Whole sample *N =* 296	Not available (missing)	Cyst infection *n =* 21	No cyst infection *n* = 275
Recipient characteristics				
Age – yr	60 (8)	0	63 (4)	56 (10)
Male	157 (53%)	0	10 (48%)	147 (53%)
History of diabetes	27 (9%)	0	4 (19%)	23 (8%)
History of cardiovascular disease	107 (36%)	0	8 (38%)	99 (36%)
History of nephrectomy (uni- or bilateral)	46 (16%)	0	3 (14%)	43 (16%)
History of cyst infection	43 (15%)	0	7 (33%)	36 (13%)
BMI (kg/m^2^)	26 (4)	0	26 (4)	26 (2)
HLA sensitization class I	107 (40%)	26	7 (41%)	100 (40%)
HLA sensitization class II	84 (31%)	26	3 (19%)	81 (32%)
Donor characteristics				
Living donor	61 (21%)	0	6 (29%)	55 (19%)
Deceased donor	235 (79%)	0	15 (71%)	220 (80%)
Expanded criteria donor	126 (43%)	0	10 (48%)	116 (42%)
Transplantation characteristics				
Retransplantation	37 (13%)	0	0 (0%)	37 (13%)
Pre-emptive transplantation	114 (39%)	0	10 (48%)	104 (38%)
Cold ischemia (hours)	12 (8)	0	13 (9)	12 (7)
Delayed graft function	138 (47%)	0	13 (62%)	125 (45%)
Induction regimen				
Thymoglobulin antibodies	139 (47%)	0	8 (38%)	131 (48%)
Anti-CD25	154 (52%)	0	13 (62%)	141 (51%)
None	3 (1%)	0	0 (0%)	3 (1%)
Steroid-free regimen	63 (22%)	6	2 (10%)	61 (23%)
Steroids exposure ≥ 1 years	116 (40%)	6	11 (52%)	105 (39%)
Transplantation follow-up				
Urinary tract infection^a^	74/296 (25%)	0	4/21 (19%)	70/275 (25%)
eGFR at 3 mo after kidney transplantation (ml/min per 1.73 m^2^)	44 (16)	7	43 (18)	44 (5)

ADPKD, autosomal dominant polycystic kidney disease; BMI, body mass index; eGFR, estimated glomerular filtration rate; HLA, human leukocyte antigen; MDRD, modification of diet in renal disease.

Continuous variables were summarized as mean and SD, and categorical and ordinal variables were summarized as frequencies and percentages.

a Excluding renal cyst infection.

By the end of the follow-up period, 29 patients (10%) had died with functional graft, and 18 (6%) had graft failure (return to dialysis or preemptive retransplantation). Sixteen patients in our cohort were lost to follow-up (median follow-up time 4.12 [2.06–6.97] years). Overall, the median follow-up time was 4.04 [2–7.01] years.

### Cyst Infection Incidence

**Figure 2:**
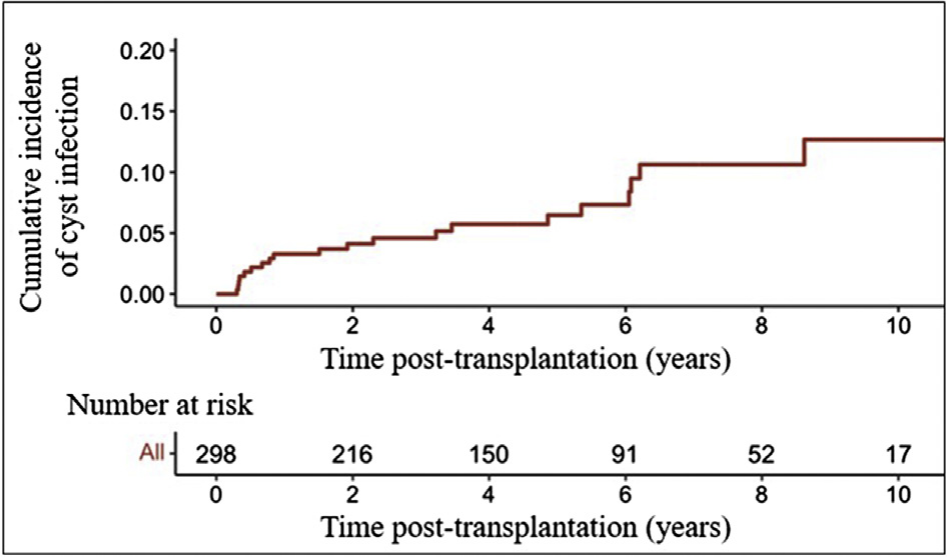
Cumulative incidence curve of cyst infection of patients with ADPKD and functional kidney allografts. ADPKD, autosomal dominant polycystic kidney disease.

A total of 21 patients (7%) experienced at least 1 cyst infection during the post-transplantation period. Cumulative incidence rate at 1, 5, and 10 years after transplantation was 3%, 6%, and 12 %, respectively, and the incidence rate was 1.6 episodes per 100 person-years (Figure 2). Median time between renal transplantation and the occurrence of the first cyst infection was 23 (6–65) months, and 9 episodes (41%) occurred within the first year after transplantation.

### Cyst Infection Characteristics at Diagnosis

A total of 21 patients experienced 22 episodes of cyst infection during the follow-up; 2 (9%) were definite, and 20 (91%) were probable. A total of 7 episodes (32%) had atypical presentation such as isolated fever (*n* = 5, 23%) (Table 2). Acute kidney injury occurred in 12 episodes (55%), mostly Acute Kidney Injury Network stage 1 (*n* = 6, 50%). Severe sepsis or septic shock were observed in 3 (14%) cyst infection episodes.

**Table 2:** Main characteristics at diagnosis of native kidney or liver cyst infection in 21 patients ADPKD with functional renal allografts at diagnosis

	Kidney transplant patient *n =* 21/episodes = 22
Clinical features	
Age-yr	61 (9)
Male	10/21 (48%)
Atypical clinical signs	7 (32%)
Lack of fever	2 (9%)
Isolated fever	5 (23%)
Severe sepsis/septic shock	3 (14%)
Laboratory findings	
Leukocyte (G/l)	8.3 (4)
Polynuclear neutrophils (G/l)	5.5 (2.6)
C-reactive protein (mg/l)	161 (29)
Acute kidney injury	12 (55%)
AKIN 1	6/12 (50%)
AKIN 2	2/12 (17%)
AKIN 3	4/12 (33%)
Positive imaging exams	
Ultrasound	3/19 (16%)^a^
CT scan	5/12 (33%)^a^
PET-CT	14/16 (87%)
Location of cyst infection	19 (86%)
Native kidney	12/19 (63%)
Liver	7/19 (37%)
Microbiological features	
*Positive culture*	16 (73%)
Cyst fluid	2^b^
Blood culture	8^b^
Urine culture	9^b^
Microbial identification	
Gram-negative bacilli	13/16 (81%)
*Escherichia coli*	8 (50%)
Others^c^	5 (31%)
Gram positive bacteria^d^	4/16 (25%)
*Enterococcus faecalis*	2 (13%)
*Streptococcus* species	2 (13%)

ADPKD, autosomal dominant polycystic kidney disease; CT, computed tomography; PET, positron emission tomography.

Continuous variables were summarized as mean and SD, and categorical and ordinal variables were summarized as frequencies and percentages.

a In 2 episodes with positive ultrasound features and 1 episode with positive CT scan features for cyst infection, a PET-CT was also performed, confirming the location of cyst infection.

b Two patients had both positive blood and urine culture, and 1 patient had both positive cyst fluid and blood culture.

c *Morganella morganii n =*1, *Klebsiella oxytoca n =*1, *Pseudomonas aeruginosa n =* 1, *Bacillus licheniformis* and *Enterobacter cloacae n =* 1

d One patient had multibacterial cyst infection with both *Escherichia.coli* and *Enterococcus faecalis*.

The imaging identified anatomical localization of the cyst infection in 19 episodes (86%) using PET-CT (*n* = 14), CT scan (*n* = 4), or renal ultrasound (*n* = 1) (Table 3). Native kidney cyst infection occurred in 12 of 19 episodes (63%) and liver cyst infection in 7 of 19 episodes (37%). Patients with liver cyst infection were older (64 ± 4 vs. 58 ± 7 years, *P* = 0.023), with a slight female predominance (5 of 6 [63%] vs. 3 of 12 [25%], *P* = 0.043) than patients with native kidney cyst infection (Table 3).

**Table 3:** Main characteristics at diagnosis of cyst infection in 18 patients with ADPKD with functional allograft according to the location of cyst infection on imaging exams

	Native kidney cyst infection *n =* 12/episode *n =* 12	Liver cyst infection *n =* 6/episode *n =* 7	*P* Value
Clinical features			
Age, yr	58 (7)	64 (4)	0.023
Male	9/12 (75%)	1/6 (17%)	0.043
Delay between transplantation and cyst infection (mo)	35 (36)	37 (25)	0.8
Pre-emptive transplantation	6/12 (50%)	4/6 (67%)	0.64
Atypical clinical signs	4 (33%)	3 (43%)	1
Lack of fever	1 (8%)	1 (14%)	1
Isolated fever	3 (25%)	2 (29%)	1
Severe sepsis/septic shock	1 (8%)	2 (29%)	0.52
Laboratory findings			
Leukocyte (G/l)	8.5 (4.2)	7.9 (3.8)	0.76
Polynuclear neutrophils (G/l)	6.8 (3.5)	6.1 (3.4)	0.71
C-reactive protein (mg/l)	150 (75)	184 (62)	0.31
Acute kidney injury	7 (58%)	5 (71%)	0.66
AKIN 1	3 (43%)	3 (60%)	1
AKIN 2	1 (14%)	1 (20%)	1
AKIN 3	3 (43%)	1 (20%)	0.58
Microbiological features			
Positive culture	10 (83%)	3 (43%)	0.13
Cyst fluid	1	1	
Blood culture	4	3	
Urine culture	6	—	
Microbial identification			
Gram-negative bacilli	7 (70%)	3 (100%)	0.53
*Escherichia coli*	4 (40%)	1 (33%)	1
Others	3 (30%)^a^	2 (67%)^b^	0.51
Gram positive bacteria	3 (30%)	0 (0)	0.53
*Enterococcus faecalis*	1 (10%)	—	
*Streptococcus* species	2 (20%)	—	

ADPKD, autosomal dominant polycystic kidney disease.

Continuous variables were summarized as mean and SD, and categorical and ordinal variables were summarized as frequencies and percentages.

a *Morganella morganii n =*1, *Pseudomonas aeruginosa n =* 1 and *Bacillus licheniformis n =* 1.

b *Klebsiella oxytoca n =*1 and *Enterobacter cloacae n =* 1

A microorganism was found in 16 (73%) cyst infection cases. Gram-negative bacilli (*n* = 13 of 16, 81%) with *Escherichia coli* (*n* = 8 of 16, 50%) were most common. Multibacterial cyst infection was observed in 1 episode (5%) (*Enterococcus faecalis* and *E. coli*). Blood cultures were positive in 8 and urine culture in 9 episodes, while both blood and urine culture were positive in 2 (Table 2). There was no difference in clinical, biological, and microbiological characteristics at diagnosis between patients with early (1 year after renal transplantation) or late (>1 year) cyst infection (Supplementary Table S1).

### Cyst Infection Treatment and Outcomes

A total of 6 patients (27%) had treatment failure, and 2 (9%) had an early recurrence of cyst infection (Table 4). In 2 patients with early recurrence, 1 had *Bacillus licheniformis* treated with dual-antibiotic therapy for 35 days with recurrence 40 days after antibiotics discontinuation. The second patient had a recurrence 7 days after antibiotic therapy discontinuation and ultimately had to undergo nephrectomy for persistent infection despite broad-spectrum antibiotic therapy. No difference was observed in treatment and outcomes between patients with early or late cyst infection after renal transplantation (Supplementary Table S2).

**Table 4:** Treatment and outcome of cyst infection in 21 patients with ADPKD with functional renal allografts

	Kidney transplant patient *n =* 21/episodes =22
Treatment	
Antibiotics therapy	
Beta-lactam alone	6 (27%)
Fluoroquinolone alone	8 (36%)
Beta lactam + fluoroquinolone	3 (14%)
Beta lactam + another antibiotic^a^	4 (18%)
Fluoroquinolone + another antibiotic^b^	1 (5%)
*Antibiotic duration-d*	33 (11)
Outcomes	
Treatment failure	6 (27%)
Modification of antibiotic therapy	6
Cyst drainage^c^	1
Nephrectomy	1 (5%)
Early recurrence^d^	2 (9%)
Immunosuppression reduction following cyst infection	2 (9%)

ADPKD, autosomal dominant polycystic kidney disease.

Continuous variables were summarized as mean and SD, and categorical and ordinal variables were summarized as frequencies and percentages.

a Trimethoprim/sulfamethoxazole *n =*1, Linezolid *n=*1, Metronidazole *n =*2.

b Metronidazole *n =*1.

c One patient had both modification of antibiotic therapy and cyst drainage because of persistent cyst infection.

d Recurrence of cyst infection within 3 months after discontinuation of initial antibiotics therapy.

Treatment failure occurred in 5 of 12 (42%) native kidney cyst infection episodes and in 1 of 7 (14%) liver cyst infection episodes (*P* = 0.33). Early recurrence was observed in 2 of 12 (17%) kidney cyst infection and in no liver cyst infection episodes (*P* = 0.51).

A total of 4 patients with Gram-negative cyst infection underwent colonoscopy; 2 had benign polyps, 1 had uncomplicated diverticulosis, and 1 had normal colonoscopy. None of the recruited patients had a bladder evaluation or commenced prophylactic antibiotic after cyst infection.

### Risk Factors for Cyst Infection

In multivariate analysis, history of cyst infection (before transplantation) was associated with the occurrence of cyst infection after renal transplantation (HR 3.47, 95% CI 1.29–9.31, *P* = 0.014). In contrast, neither induction treatment with thymoglobulin antibodies, nor steroids use in the follow-up period, nor steroids exposure ≥ 1 year was associated with cyst infection (Table 5).

**Table 5:** Factors associated with post transplantation native kidney or liver cyst infection in univariate and multivariate analyses

Exposure	Univariate HR (95 % CI)	Univariate *P* value	Multivariate HR (95% CI)	Multivariate *P* value
Recipient characteristics				
Age	1.03 (0.98–1.08)	0.27	1.02 (0.97–1.07)	0.49
Male	0.81 (0.34–1.91)	0.64	—	
History of diabetes	2.32 (0.67–7.94)	0.18	1.08 (0.14–8.52)	0.94
History of cardiovascular disease	1.18 (0.47–2.94)	0.73	—	
History of unilateral nephrectomy	0.99 (0.29–3.38)	0.99	—	
History of cyst infection	2.67 (1.07–6.63)	0.034	3.47 (1.29–9.31)	0.014
BMI (kg/m^2^)	1.02 (0.91–1.13)	0.75	—	—
Transplantation characteristics at baseline				
Cadaveric donor	0.68 (0.26–1.76)	0.42	—	—
Standard criteria donor	0.56 (0.19–1.64)	0.29	—	—
Pre-emptive transplantation	1.35 (0.57–3.18)	0.5	—	—
Cold ischemia (h)	1.00 (0.95–1.06)	0.99	—	—
Delayed graft function	0.51 (0.12-2.19)	0.36	—	—
Immunosuppression regimen				
Thymoglobulin antibodies	0.88 (0.37–2.08)	0.77	—	—
Steroid-free regimen	0.87 (0.29–2.62)	0.81	—	—
Steroids exposure ≥ 1 yr	1.38 (0.53–3.57)	0.51	1.19 (0.14–8.52)	0.73
Transplantation follow-up				
eGFR at 3 mo	0.99 (0.96–1.02)	0.67	—	—
Urinary tract infection^a^	0.82 (0.30–2.25)	0.70	—	—

ADPKD, autosomal dominant polycystic kidney disease; BMI, body mass index; eGFR, estimated glomerular filtration rate; HR, hazard ratio; MDRD, modification of diet in renal disease.

a Excluding renal cyst infection.

In subgroup multivariable analysis of patients with only imaging-proven native kidney cyst infection (*n =* 12), history of cyst infection remained associated with the occurrence of cyst infection during the post-transplantation period (HR 2.92, 95% CI 1.17–7.29, *P* = 0.022) (Supplementary Table S3).

Of note, pretransplantation prophylactic nephrectomy was not associated with the incidence of cyst infection (HR 0.99, 95% CI 0.29–2.38, *P* = 0.99). In patients with a history of cyst infection (*n* = 43), 1 of 9 patients (9%) with prophylactic unilateral nephrectomy had imaging-proven renal cyst infection versus 4 of 31 patients (13%) who did not underwent prophylactic nephrectomy (*P* = 0.99) (Figure 1). Thus, 1 patient developed a left native kidney cyst infection (diagnosed with FDG-PET-CT) 9 years after renal transplantation despite a right nephrectomy for right renal cyst infection recurrence done 19 months before renal transplantation.

### Association Between Cyst Infection and Death-Censored Graft Survival

A total of 5 patients (24%) in cyst infection group and 13 patients (5%) in the noncyst infection group had death-censored graft failure. The time between cyst infection and death-censored graft failure was 2, 4, 7, 17, and 104 months, respectively. In multivariable analysis, cyst infection was significantly associated with death-censored graft failure (HR 3.93, 95% CI 1.21–12.80, *P* = 0.023) (Supplementary Table S4). The 2 patients with cyst infection 2 and 4 months before graft failure had severe renal insufficiency (estimated glomerular filtration rates at 20 and 14 ml/min per 1.73 m^2^, respectively) already before their cyst infection episode. Among the 3 others, 2 had alternative causes of graft failure (chronic antibody-mediated rejection after reduced immunosuppression after severe legionellosis and acute respiratory distress syndrome because of *Pneumocystis jirovecii* and immunosuppression withdrawal, respectively); the last had cyst infection and early recurrence with a concomitant decline of renal function in association with chronic heart failure and returned to dialysis 7 months after the cyst infection episode (Supplementary Figure S1).

## Discussion

In our large cohort of kidney transplant recipients with ADPKD, the incidence of cyst infection after renal transplantation was 1.6 per 100 person-years, and the only predictive factor of cyst infection was a history of cyst infection before renal transplantation. In one-fifth of cases, cyst infection in renal transplant patients presented as isolated fever and was frequently associated with acute kidney injury.

To best of our knowledge, this study is the first to investigate specifically cyst infection in patients with ADPKD with renal allograft. Sallée *et al.*^11^ identified retrospectively cyst infection in patients with ADPKD with or without renal replacement therapy using a single-center computerized patient database and estimated an incidence rate of 1 per 100 person-years, which is consistent with our data. We found that more than one-third of cyst infection occurred within the first year after renal transplantation, which may reflect more intense immunosuppression given the first year after transplantation. Interestingly, we did not find any association between induction regimen or steroid exposure and the occurrence of cyst infection. However, urinary tract infections such as graft pyelonephritis occurrence have not been associated with induction regimen or steroids exposure in previous studies either.^19^, ^20^, ^21^ Because renal cyst infection are generally associated with higher kidney volume,^22^ it is possible that the natural decrease in native kidney volume, observed mainly within the first year after renal transplantation,^23^ also played a role in renal cyst infection after the first year of transplantation.

Using a large cohort of patients with ADPKD, we identified an association between history of cyst infection and cyst infection occurrence during the post-transplantation period. This finding held true even after a more stringent subgroup analysis including only radiologically proven native kidney cyst infection. The previous studies investigating risk factors of cyst infection in ADPKD were either underpowered or based on very limited clinical data. Only 1 retrospective case series of 50 patients with ADPKD on chronic dialysis suggested an association between predialysis history of cyst infection and the occurrence of renal cyst infection during dialysis period (not a statistically significant finding).^12^ Without much reliable evidence, other cyst infection risk factors such as female sex, age, and recent instrumentation of the urinary tract or contaminant urine or biliary infection have been proposed.^13^ We did not find any association between age, sex, or urinary tract infection with cyst infection occurrence in our cohort.

Our finding could have an important clinical implication because prophylactic nephrectomy still remains controversial in the peritransplantation period of patients with ADPKD.^9^ The indication such prophylactic procedure must consider (i) surgical complication rate,^24^^,^^25^ which is, at least in part, influenced by the local operator experience^26^; (ii) cyst infection incidence after transplantation (in our cohort, most of kidney transplant patients with a history of cyst infection did not experience cyst infection after a median follow-up of 4 years); and (iii) the fact that despite prophylactic unilateral nephrectomy for cyst infection pretransplantation recurrence, cyst infection could still occur in the contralateral native kidney after transplantation as shown in 1 patient in our study. Renal cyst infection is a rare event after renal transplantation even in patients with a history of cyst infection, and so, prophylactic pretransplantation nephrectomy should be reserved only for patients with frequent renal cyst infection recurrence or life-threatening cyst infection. Because only 11 patients with previous cyst infection had unilateral native nephrectomy before renal transplantation, our study was not sufficiently powered to explore its protective effect on renal cyst infection.

Cyst infection diagnosis is particularly challenging in immunocompromised patients, as the typical clinical signs of infection could be missing.^27^ In our cohort, almost one-quarter of patients had isolated fever, making FDG-PET-CT particularly useful for diagnosis of cyst infection. This is consistent with previous reports highlighting the role of FDG-PET-CT in isolated fever or inflammation of unknown origin in patients with ADPKD for the diagnosis of cyst infection and to rule out alternative diagnoses such as sigmoiditis.^15^^,^^28^ Similar to reports on nontransplanted patients with ADPKD, *E. coli* was the most common bacteria cultured in our cyst infection cohort.^11^^,^^29^^,^^30^ Although not statistically significant, *E. coli* seemed less frequently involved in late cyst infection (>1 year after renal transplantation), when broader Gram-negative bacteria species such as cephalosporinase-inducible or nonfermenting Gram-negative bacilli were found more often. Thus, in late cyst infection, empirical antibiotic therapy with a third-generation cephalosporin may be ineffective, and cyst aspiration or drainage should be considered.

Finally, because bacterial infection may trigger alloimmune response,^31^ we wondered whether cyst infection episodes could impact the graft survival. Although cyst infection was significantly associated with death-censored graft failure, alternative causes were found in almost all cases: severe infection such as legionellosis, occurrence of de novo donor specific antibodies and chronic humoral rejection after immunosuppression wean, severe pneumocystosis inducing graft failure 3 years after cyst infection, or pre-existing severe renal impairment before the cyst infection occurrence (Supplementary Figure S1). Thus, we could not establish a definite causal relationship between the occurrence of the cyst infection and the graft loss.

Our study has several limitations. First, because clinical presentation of acute graft pyelonephritis and native renal cyst infection is very similar, we might have underestimated the cyst infection incidence, in particular when patient with cyst infection were misdiagnosed with acute graft pyelonephritis only based on fever and leukocyturia. In contrast, we used previously published and commonly used clinical criteria for cyst infection to minimize this bias. Second, because of the monocentric study design, the number of cyst infection events detected was limited and may have reduced statistical power to detect other potential risk factors. Third, in some cases, we could not confirm the cyst infection location (liver vs. renal) before transplantation and in the post-transplantation period and whether each cyst infection episode before transplantation was fulfilling cyst infection criteria. Finally, as numerous patients underwent prophylactic nephrectomy before transplantation, this may have decreased the incidence of cyst infection in the post-transplantation period.

Although uncommon, diagnosis of cyst infection after renal transplantation can be challenging and occasionally lead to severe sepsis. In our cohort, cyst infection rate did not seem affected by immunosuppression given for renal transplantation. The pretransplantation history of cyst infection represented the most significant risk for cyst infection after transplantation. Further studies with larger number of patients will be needed to evaluate the long-term outcomes and additional risk factor for cyst infection in kidney transplant recipients.

## Disclosure

All the authors declared no competing interests.

## Supplementary Material

Supplementary File (WORD)

**Figure S1.** Timeline showing cyst infection and graft failure. Tx, transplantation; Pt, patient; eGFR, estimated glomerular filtration ml/min/1.73m^2^ (MDRD); CyI, cyst infection; ARDS, acute respiratory distress syndrome; RD, return to dialysis; nDSA, de novo donor specific antibodies; cABMR, chronic active antibodies mediated rejection**.**

**Table S1.** Main characteristics of cyst infection in 21 patients with ADPKD with functional renal allografts at diagnosis according to early (1 year) or late (>1 year) onset of cyst infection after transplantation.

**Table S2.** Treatment and outcomes of 21 patients with ADPKD with functional renal allografts at diagnosis according to early (<1 year) or late (>1 year) onset of cyst infection after transplantation**.**

**Table S3.** Factors associated with radiologically proven native kidney cyst infection post transplantation using univariate and multivariate analyses.

**Table S4.** Risk factors of death censored graft survival in univariate and multivariate analysis.
