# Supplementary material for: Incidence, Risk Factors and Outcomes of Kidney and Liver Cyst Infection in Kidney Transplant Recipient With ADPKD
Source: Kidney Int Rep. 2022 Feb 3;7(4):867–75. doi: 10.1016/j.ekir.2022.01.1062 (PMC9039903; doi:10.1016/j.ekir.2022.01.1062)
Supplement: Supplementary File (WORD) [file mmc1.docx]

|  | **Early cyst infection < 1 year after kidney transplantation**  **n= 9/episode n =9** | **Late cyst infection** $\boldsymbol{\geq}$**1 year after kidney transplantation**  **n= 12 /episode n =13** | **P**  **Value** |
| --- | --- | --- | --- |
| **Clinical features**  Age -years  Male  Delay between transplantation and cyst infection (months)  *Transplantation characteristics*  Preemptive transplantation  Induction regimen  Antithymocyte globulin  Anti CD25  *Maintenance regimen at time of cyst infection*  Cyclosporine  Tacrolimus  mTor inhibitor  Mycophenolate mofetil/mycophenolic acid  Azathioprine  Steroids  *Atypical clinical signs*  Lack of fever  Isolated fever  *Severe sepsis/septic shock*  **Laboratory findings**  Leukocyte (G/L)  Polynuclear neutrophils (G/L)  C-reactive protein (mg/L)  Acute kidney injury  AKIN 1  AKIN 2  AKIN 3  Microbiological features  *Positive culture*  Cyst fluid  Blood culture  Urine culture  *Microbial identification*  Gram negative bacilli  E.coli  Others  Gram positive bacteria  Enterococcus faecalis  Streptococcus species | 57 (10)  4/9 (44%)  6 (3)  4/9 (44%)  4/9 (44%)  5/9 (66%)  0 (0)  8 (89%)  1 (11%)  6 (67%)  0 (0)  6 (67%)  3 (33%)  1 (11%)  2 (22%)  0 (0)  6.4 (2.6)  4.7 (1.8)  165 (62)  6 (67%)  3 (50%)  1 (17%)  2 (33%)  5 (55%)  0  3  4  4/5 (80%)  4 (80%)  0 (0)  1/5 (20%)  1 (20%)  0 (0) | 64 (7)  6/12 (50%)  59 (33)  6/12 (50%)  4/12 (33%)  8/12 (58%)  2 (15%)  11 (85%)  0 (0)  7 (54%)  1 (7%)  4 (31%)  4 (31%)  1 (8%)  3 (23%)  3 (23%)  8.9 (4.1)  7.2 (3.6)  159 (75)  6 (46%)  3 (50%)  1 (17%)  2 (33%)  11 (85%)  2  5  5  9/11 (82%)  4 (36%)  5 (45%) **  3/11 (27%)  1 (9%)  2 (18%) | 0.15  1  <0.001  1  0.67  1  0.49  1  0.41  0.67  1  0.19  1  1  1  0.2  0.1  0.08  0.84  0.49  0.66  1  1  0.18  1  0.28  0.12  1  1  1 |

**Supplemental Table S1.** **Main characteristics of cyst infection in 21 ADPKD patients with functional renal allografts at diagnosis according to early (**$\boldsymbol{\leq}$**1year) or late (**$\boldsymbol{>}$ **1 year) onset of cyst infection after renal transplantation.**

Continuous variables were summarized as mean and standard deviation (SD), and categorical and ordinal variables were summarized as frequencies and percentages.

**) Morganella morganii n =1, Klebsiella oxytoca n =1, Pseudomonas aeruginosa n = 1, Bacillus Licheniformis and Enterobacter cloacae n = 1

|  | **Early cyst infection < 1 year after kidney transplantation**  **n= 9/episode n =9** | **Late cyst infection** $\boldsymbol{\geq}$**1 year after kidney transplantation**  **n= 12 /episode n =13** | **P value** |
| --- | --- | --- | --- |
| **Treatment**  *Antibiotics therapy*  Beta-lactam alone  Fluoroquinolone alone  Beta lactam + fluoroquinolone  Beta lactam + another antibiotic *  Fluoroquinolone + another antibiotic**  *Antibiotics duration-days*  **Outcomes**  Treatment failure  Modification of antibiotic therapy  Cyst drainage  Nephrectomy  Early recurrence  Immunosuppression reduction following CI | 3 (33%)  4 (44%)  0 (0)  1 (11%)  1 (11%)  32 (9)  3 (33%)  3 (33%)  0 (0)  1 (11%)  1 (11%)  1 (11%) | 3 (23%)  4 (31%)  3 (23%)  3 (23%)  0 (0)  33 (12)  3 (23%)  3 (33%)  1 (11%)  0 (0)  1 (8%)  1 (8%) | 0.66  0.66  0.24  0.61  0.41  0.94  0.66  0.66  1  0.41  1  1 |

**Supplemental Table S2.** **Treatment and outcomes of 21 ADPKD patients with functional renal allografts at diagnosis according to early (**$\boldsymbol{\leq}$**1year) or late (**$\boldsymbol{>}$ **1 year) onset of cyst infection after renal transplantation.**

Continuous variables were summarized as mean and standard deviation (SD), and categorical and ordinal variables were summarized as frequencies and percentages.

*) Trimethoprim/sulfamethoxazole n =1, Linezolid n=1, Metronidazole n =2.

**) Metronidazole n =1.

|  | **Univariate**  **HR (95 % CI)** | **Univariate**  **P value** | **Multivariate**  **HR (95% CI)** | **Multivariate**  **P value** |
| --- | --- | --- | --- | --- |
| **Recipient characteristics ^§^**  Age  Male  History of diabetes  History of cardio-vascular disease  History of unilateral nephrectomy  History of cyst infection  BMI (kg/m²)  **Transplantation characteristics at baseline**  Cadaveric donor  Standard criteria donor  Pre-emptive transplantation  Cold ischemia (hours)  Delayed graft function  **Immunosuppression regimen**  Thymoglobulin antibodies  Steroid free regimen  Steroids exposure $\geq$1 year  **Transplantation follow-up**  Upper Urinary tract infection*  eGFR at 3 months post transplantation | 1.03 (0.98 to 1.08)  0.82 (0.35 to 1.94)  2.30 (0.67 to 7.89)  1.17 (0.47 to 2.92)  0.98 (0.29 to 3.35)  2.78 (1.12 to 6.90)  1.02 (0.92 to 1.13)  0.68 (0.26 to 1.77)  0.56 (0.19 to 1.67)  1.33 (0.56 to 3.15)  1.00 (0.95 to 1.06)  0.50 (0.12-2.17)  0.87 (0.37 to 2.05)  0.86 (0.29 to 2.60)  1.36 (0.52 to 3.52)  0.82 (0.30 to 2.25)  0.99 (0.96 to 1.02) | 0.27  0.66  0.19  0.74  0.98  0.028  0.71  0.43  0.3  0.51  0.99  0.36  0.75  0.80  0.53  0.70  0.67 | -  0.81 (0.34 to 1.93)  2.71 (0.77 to 9.52)  -  -  2.92 (1.17 to 7.29)  -  -  -  -  -  -  -  -  -  -  - | -  0.63  0.12  -  -  0.022  -  -  -  -  -  -  -  -  -  -  - |

**Supplemental Table S3.** **Factors associated with post transplantation imaging proven native kidney cyst infection in univariate and multivariate analyses.**

BMI, body mass index; eGFR, estimated glomerular filtration rate (MDRD, modification of diet in renal disease ); 95 % CI, 95 % confidence interval

§) excluding the 4 patients who underwent bilateral prophylactic nephrectomy before kidney transplantation

*) excluding renal cyst infection

|  | **Univariate**  **HR (95 % CI)** | **Univariate**  **P value** | **Multivariate**  **HR (95 % CI)** | **Multivariate**  **P value** |
| --- | --- | --- | --- | --- |
| **Recipient characteristics**  Age  Male  History of diabetes  History of cardio-vascular disease  History of unilateral nephrectomy  History of cyst infection  BMI (kg/m²)  **Transplantation characteristics at baseline**  Pre-emptive transplantation  Cadaveric donor  Standard criteria donor  Cold ischemia (hours)  Delayed graft function  **Immunosuppression regimen**  Thymoglobulin antibodies  Steroid free regimen  Steroid’s exposure $\geq$1 year  **Follow up data**  Rejections  De novo DSA  Cyst infection | 1.05 (1.00 to 1.10)  1.30 (0.52 to 3.24)  3.72 (1.21 to 11.37)  2.80 (1.13 to 6.97)  0.73 (0.17 to 3.19)  0.95 (0.28 to 3.25)  1.00 (0.89 to 1.12)  0.62 (0.24 to 1.65)  2.27 (0.52 to 9.85)  0.63 (0.23 to 1.70)  1.06 (1.00 to 1.00)  5.04 (1.88 to 13.54)  0.44 (0.17 to 1.16)  0.97 (0.32 to 2.97)  1.43 (0.55 to 3.70)  2.42 (0.87 to 6.75)  3.30 (1.06 to 10.27)  3.64 (1.30 to 10.16) | 0.069  0.57  0.021  0.026  0.68  0.929  0.96  0.34  0.28  0.36  0.045  0.001  0.098  0.96  0.47  0.091  0.040  0.014 | 1.06 (1.00 to 1.13)  -  -  -  -  -  -  -  -  -  -  5.16 (1.83 to 14.59)  -  -  -  3.21 (1.08 to 9.54)  -  3.93 (1.21 to 12.80) | 0.064  -  -  -  -  -  -  -  -  -  -  0.002  -  -  -  0.036  -  0.023 |

**Supplemental Table S4.** **Risk factors of death censored graft survival in univariate and multivariate analysis.**

BMI, body mass index; DSA, donor specific antigen; 95 % CI, 95 % confidence interval

**Supplemental Figure S1. Timeline relation between cyst infection and graft failure.**


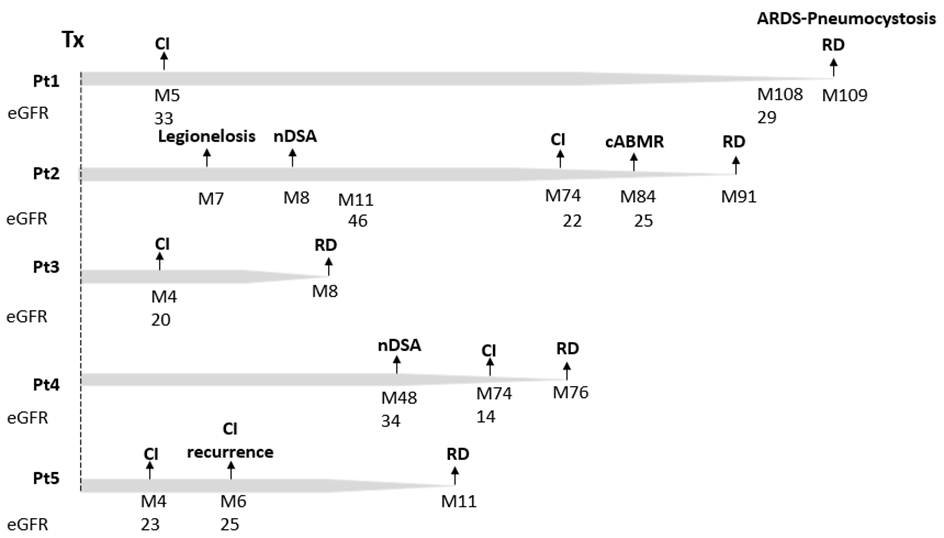


Tx, transplantation; Pt, patient; eGFR, estimated glomerular filtration ml/min/1.73m² (MDRD, modification of diet in renal disease); CyI, cyst infection; ARDS, acute respiratory distress syndrome; RD, return to dialysis; nDSA, de novo donor specific antibodies; cABMR, chronic active antibodies mediated rejection.
